# Sustainably Synthesized CeO_2_ Nanoparticles from Lemon Juice and Sucrose for Antibacterial Applications

**DOI:** 10.3390/mi17070760

**Published:** 2026-06-23

**Authors:** Matilde Carvalho, Susana Devesa, Daniela Santo, Sandra Carvalho, Zohra Benzarti

**Affiliations:** 1 University of Coimbra, Physics Department, Rua Larga, 3004-516 Coimbra, Portugal; matildecarvalho690@gmail.com; 2 CEMMPRE—Centre for Mechanical Engineering, Materials and Processes, ARISE—Advanced Production and Intelligent Systems, Department of Mechanical Engineering, University of Coimbra, 3030-788 Coimbra, Portugal; dsanto@dem.uc.pt (D.S.); sandra.carvalho@dem.uc.pt (S.C.); 3 IPN—LED&MAT—Instituto Pedro Nunes, Laboratório de Ensaios, Desgaste e Materiais, Rua Pedro Nunes, 3030-199 Coimbra, Portugal

**Keywords:** green synthesis, cerium oxide nanoparticles, lemon juice, sucrose, antibacterial activity

## Abstract

Green synthesis of metal oxide nanoparticles is a promising route to reduce toxic reagents and energy consumption while enabling biocompatible nanomaterials for biomedical use. In this work, cerium oxide (CeO_2_) nanoparticles were synthesized using lemon juice and sucrose as bio-based chelating, capping and stabilizing agents. Three synthesis routes were designed by varying the use of lemon juice, sucrose, or their combination. The synthesized materials were characterized using thermal analysis (DSC—Differential Scanning Calorimetry and TGA—Thermogravimetric Analysis), X-ray diffraction (XRD), Raman spectroscopy, and scanning electron microscopy (SEM). Additionally, their antibacterial activity was assessed against Gram positive bacterium *Staphylococcus aureus* (*S. aureus*). Thermal analysis showed that heat treatment at 600 °C promotes high crystallinity, as evidenced by the development of sharp diffraction peaks associated with the cubic fluorite CeO_2_ structure, and a dominant F_2g_ Raman mode at 463 cm^−1^. SEM micrographs revealed nanometric particles and highlighted that combining lemon juice and sucrose effectively suppresses coalesced structures, yielding more homogeneous morphologies. Crystallite size calculations gave average sizes of 17.2 nm, with the lemon juice-only route producing the largest crystallites. Antibacterial tests revealed a clear dose-dependent inhibition of *S. aureus*, with marked inhibition of bacterial growth at concentrations ≥5 mg/mL and a plateau effect above 25 mg/mL. This study confirms the feasibility of using plant-based extracts as sustainable reagents for CeO_2_ nanoparticle synthesis, with promising structural and biological performance for potential biomedical applications.

## 1. Introduction

Nanotechnology has enabled the design of nanomaterials with size dependent physicochemical properties that are unattainable in their bulk counterparts, opening opportunities in biomedicine, catalysis, environmental remediation, and energy systems [1,2,3,4]. Metal and metal oxide nanoparticles are particularly attractive due to their high surface-to-volume ratio, tunable surface chemistry, and versatile optical, electronic, and catalytic behavior, which can be engineered through precise control of size, morphology, and crystallinity [5,6]. However, conventional synthetic routes rely on harsh conditions, toxic precursors, and energy-intensive processing, raising concerns about environmental impact and biocompatibility in biomedical applications [7].

Green synthesis has therefore emerged as a sustainable alternative that aligns with the principles of green chemistry by minimizing hazardous reagents and reducing waste [8]. In this approach, bio-based materials serve as chelating, stabilizing, and capping agents, offering a cost-effective and nontoxic platform for nanoparticle production [9,10]. Compared with purely chemical methods, plant-mediated syntheses frequently improve biocompatibility through the adsorption of functional phytochemicals onto the nanoparticle surface, which is advantageous for antimicrobial applications [11]. Recent studies have highlighted citrus-derived extracts as particularly promising green reagents, since peels and juices are rich in phenolic compounds, flavonoids, and organic acids capable of driving nucleation and growth of metal oxide nanoparticles while valorizing agri-food residues [12,13].

Among metal oxides, cerium oxide (CeO_2_) has attracted growing interest due to its redox behavior associated with the Ce^3+^/Ce^4+^ couple and its high density of oxygen vacancies [14]. These characteristics confer enzyme-mimetic catalytic activities, including superoxide dismutase- and catalase-like behavior, enabling CeO_2_ nanoparticles to scavenge reactive oxygen species (ROS) [15,16,17]. Conversely, under specific physicochemical and environmental conditions, CeO_2_ nanoparticles may also exhibit oxidase- and peroxidase-like activities, promoting ROS generation and contributing to their antibacterial properties [18]. In the last few years, a variety of plant-mediated strategies have been reported for the synthesis of CeO_2_ nanoparticles using fruit juices and peels, including watermelon and different citrus species, with demonstrated antibacterial or antioxidant performance [19,20,21,22]. Nevertheless, most works focus either on a single type of extract or on generic citrus peels, with limited systematic comparison of how specific bio-based additives and their combinations influence CeO_2_ phase formation, crystallinity, and morphology.

Lemon juice is an abundant, low-cost source of citric acid and polyphenols that simultaneously act as chelating [23] and complexing agents [24]. Similarly, sucrose is a widely available carbohydrate that can function as a benign capping and structure-directing agent in green syntheses [23,24].

Although carbohydrate sugars (fructose, lactose and glucose) have been used to produce CeO_2_ nanostructures with acceptable cytocompatibility [25], the potential synergy between crude lemon juice and sucrose in a single synthesis route has not been explored in detail. Specifically, there is a need to correlate the combined action of these natural agents with the evolution of phase purity and morphology.

The novelty of this study lies in the systematic evaluation of a multi-component green precursor system. We report the synthesis of CeO_2_ nanoparticles using lemon juice (LJ), sucrose (S), and their combination (LJ+S) as bio-based chelating, capping, and stabilizing agents, followed by thermal treatments at 350 °C and 600 °C. By systematically comparing these three formulations, we elucidate how the precursor chemistry and calcination temperature govern crystallinity, secondary phase formation, and morphology using DSC/TGA, XRD, Raman spectroscopy, SEM, and Scherrer-based crystallite size analysis. To the best of our knowledge, this is the first study combining lemon juice and sucrose in a single green synthesis route for CeO_2_. The role of these bio-based reagents in controlling nanoscale structural and morphological features was examined, and antibacterial activity against *Staphylococcus aureus* was assessed for the sample exhibiting the most favorable morphology.

## 2. Materials and Methods

### 2.1. Materials

The synthesis of CeO_2_ nanoparticles involved the use of Cerium (III) Nitrate Hexahydrate (Ce(NO_3_)_3_·6H_2_O, ≥99%, St. Louis, MO, USA), sodium hydroxide (NaOH) and commercial-grade sucrose (99.7% purity). The lemons used for the preparation of the lemon juice were collected in Coimbra, Portugal, in March. All aqueous solutions were prepared using deionized ultrapure water, self-produced using ultrapure water equipment.

### 2.2. Extract Preparation and Nanoparticle Synthesis

Lemons were peeled, and the flesh was homogenized in a food processor (during 10 min) to produce a crude extract. To retain the full profile of natural chelating, capping and stabilizing agents, the unpurified whole-fruit homogenate was utilized directly for the synthesis.

Three synthesis conditions were evaluated: lemon juice alone (LJ), sucrose alone (S), and a combination of both (LJ+S). For each condition, 8.0 g (0.0184 mol) of the cerium precursor (Ce(NO_3_)_3_·6H_2_O) was used. The reaction mixtures were designed around a 1:3 molar ratio of cerium precursor to the natural stabilizing agents [25]. Specifically, the LJ sample contained 18.9 g of crude lemon extract in 100 mL of water; the S sample contained 18.9 g (0.055 mol) of sucrose in 100 mL of water; and the LJ+S sample combined 18.9 g of extract and 18.9 g of sucrose in 100 mL of water.

The prepared mixtures were heated to 60 °C under continuous magnetic stirring. The cerium precursor was gradually added over 10 min. A 20 g/L NaOH solution was subsequently added dropwise to adjust the pH until stabilization, resulting in observable changes in the viscosity and color of the systems, consistent with the formation of a viscous sol that gradually evolved into a gel-like network (Table 1). Stirring was maintained for 10 min post-stabilization, after which the mixtures were allowed to settle overnight.

**Figure 1:**
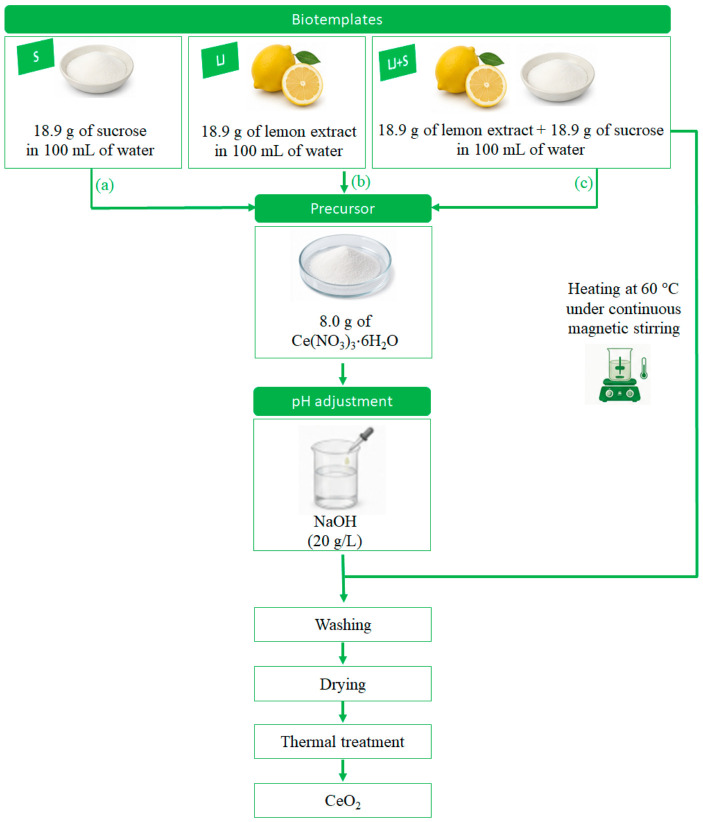
Schematic representation of the synthesis routes for the (**a**) S, (**b**) LJ, and (**c**) LJ+S samples.

The resulting materials were washed twice with 200 mL of deionized water, aided by 30 min of ultrasonication per wash. Finally, the samples were dried in an oven at 120 °C for 5 h at a heating rate of 5 °C/min. This drying cycle was performed twice for the LJ and LJ+S samples, and three times for the S sample. The green synthesis workflow is illustrated in Figure 1.

### 2.3. Characterization Techniques

Thermogravimetric analysis (TGA) was performed using a Netzsch-Gerätebau GmbH TG 209 F1 Libra (Selb, Germany) under a nitrogen atmosphere (30–1095 °C, 5 °C/min). Differential scanning calorimetry (DSC) was conducted using a Netzsch-Gerätebau GmbH DSC 204 F1 Phoenix (Selb, Germany) under nitrogen (25–500 °C, 5 °C/min).

Raman spectroscopy was performed using a Renishaw plc inVia™ (Wotton-under-Edge, UK) confocal spectrometer with a 532 nm green laser. X-ray diffraction (XRD) patterns were acquired using a Rigaku Corporation Smartlab diffractometer (Tokyo, Japan) with Cu K*α* radiation (*λ* = 1.54060 Å) operating at 40 kV and 50 mA, with Bragg-Brentano geometry and a step size of 0.02°.

Scanning electron microscopy (SEM) was performed in secondary electron mode using a Zeiss Merlin microscope (Zeiss, Oberkochen, Germany), at a magnification of 10.00 and 35.00 k× and an accelerating voltage of 1.00 kV.

### 2.4. Antibacterial Activity Assay

The antibacterial activity of CeO_2_ nanoparticles from the LJ+S sample was evaluated against the Gram-positive bacterium *Staphylococcus aureus* (ATCC 6538).

*S. aureus* cells were stored at −80 °C in Tryptic Soy Broth (TSB, Frilabo, Milheirós, Portugal) supplemented with 15% (*v*/*v*) glycerol. Prior to experimentation, the cells were streaked onto Tryptic Soy Agar (TSA, Frilabo, Milheirós, Portugal) plates and incubated at 37 °C for 36 h. A single colony was then selected, inoculated into TSB, and incubated at 37 °C for 18 h with agitation at 120 rpm. The resulting culture was diluted in TSB to achieve a final concentration of 7 × 10^6^ CFU/mL.

CeO_2_ NP suspensions were prepared at concentrations ranging from 5 to 60 mg/mL by weighing the appropriate mass of nanoparticles (10–120 mg) under sterile conditions. Subsequently, 1 mL of the bacterial suspension (7 × 10^6^ CFU/mL) was added to each well of a sterile 24-well culture plate. In parallel, 1 mL of the same bacterial suspension was added to microcentrifuge tubes containing the pre-weighed CeO_2_ NPs and thoroughly mixed to ensure homogeneous dispersion. The resulting mixtures (total volume of 2 mL) were then transferred to the corresponding wells of the 24-well plate. A control consisting of 2 mL of bacterial suspension without nanoparticles was included. Plates were incubated at 37 °C for 24 h under orbital shaking (120 rpm). The pH of the untreated control and CeO_2_ nanoparticle suspensions was measured after 24 h of incubation using a calibrated pH meter to evaluate whether changes in medium pH could contribute to the observed antibacterial effects.

Following incubation, bacterial viability was quantified using the colony-forming unit (CFU) counting method. Serial dilutions (10^−1^ to 10^−6^) were prepared in phosphate-buffered saline (PBS), and 10 µL aliquots of each dilution were plated onto TSA. After incubation at 37 °C for 24 h, visible colonies were enumerated. The bacterial concentration was calculated using the following equation [26]:

(1) $$CFU/mL=\frac{Number\;of\;colonies\times Dilution\;factor}{Volume\;plated\;(mL)}$$

All measurements were performed in duplicate, and the entire experiment was repeated in at least two independent assays to ensure reproducibility. Results were analyzed using two-way analysis of variance (ANOVA), followed by Dunnett’s multiple comparisons test to compare each treated group with the untreated control. Analyses were performed using GraphPad Prism software (GraphPad Prism 8.0.2). A *p*-value < 0.05 was considered statistically significant.

## 3. Results and Discussion

### 3.1. DSC/TGA Analysis

**Figure 2:**
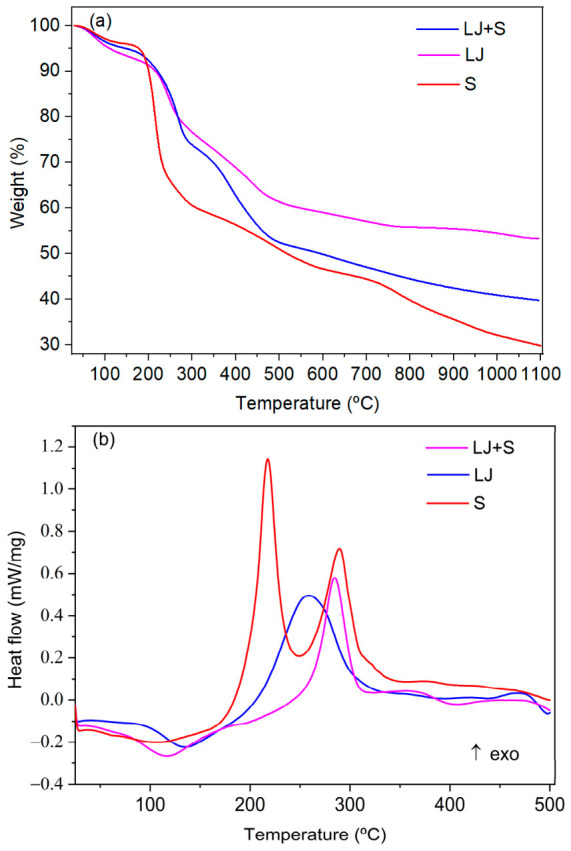
(**a**) TGA and (**b**) DSC analysis of the three samples prepared (S, LJ, and LJ+S). The upward arrow in (**b**) denotes the exothermic direction.

The thermal behavior of the S, LJ, and LJ+S samples was evaluated to identify appropriate calcination temperatures based on their thermal decomposition profiles. The DSC and TGA results are presented in Figure 2.

For the S sample, the DSC reveals a slight endothermic peak around 100 °C, which can be attributed to the evaporation of residual water and volatile organic components [27], followed by two exothermic peaks at 220–250 °C and 300 °C, indicating significant exothermic reactions, potentially linked to the formation of the CeO_2_ phase. The TGA corroborates these findings, showing an initial mass loss between 50–150 °C, followed by a substantial mass decrease between 200–400 °C, which aligns with the exothermic peaks in the DSC. Beyond this range, mass loss continues gradually up to 1000 °C, albeit at a lower rate.

In the DSC of LJ, an endothermic peak is observed between 100–150 °C, possibly associated with the loss of residual water and volatile organic components, followed by a well-defined exothermic peak at 250 °C, initially suggesting the formation of CeO_2_. However, the TGA profile indicates a continuous mass loss extending up to 600 °C, which signifies that chemical decomposition and organic burnout remain ongoing beyond this temperature. When correlated with XRD data discussed later, these results refute the hypothesis that stable, crystalline CeO_2_ is fully formed at 250 °C.

For the LJ+S sample, the DSC exhibits an initial decline at 100–150 °C, consistent with water loss, followed by an exothermic peak at 250–300 °C, suggesting the formation of CeO_2_. The TGA supports this assumption, displaying a significant mass loss between 200–400 °C, coinciding with the endothermic peak in the DSC, further reinforcing the likelihood of chemical reactions occurring within this temperature range. Above 600 °C, mass stabilization indicates that the material reaches a stable state.

Based on these observations, 350 °C was selected for the initial thermal treatment. Although mass loss continued beyond this threshold in all cases, the DSC analysis was limited to a maximum temperature of 500 °C. Consequently, 350 °C was identified as a critical point where the gradual mass loss likely corresponds to the thermal decomposition and the onset of crystallization associated with the formation of the CeO_2_ phase [28]. However, literature indicates that increasing the temperature to 600 °C is necessary to remove residual carbon-based impurities from the nanoparticle surface, although this may increase the risk of particle agglomeration [29]. The two temperatures are evaluated to determine their influence on phase crystallinity, purity, and morphology, thus establishing the justification for the selection of the sample used in subsequent biological analyses.

### 3.2. XRD Analysis

**Figure 3:**
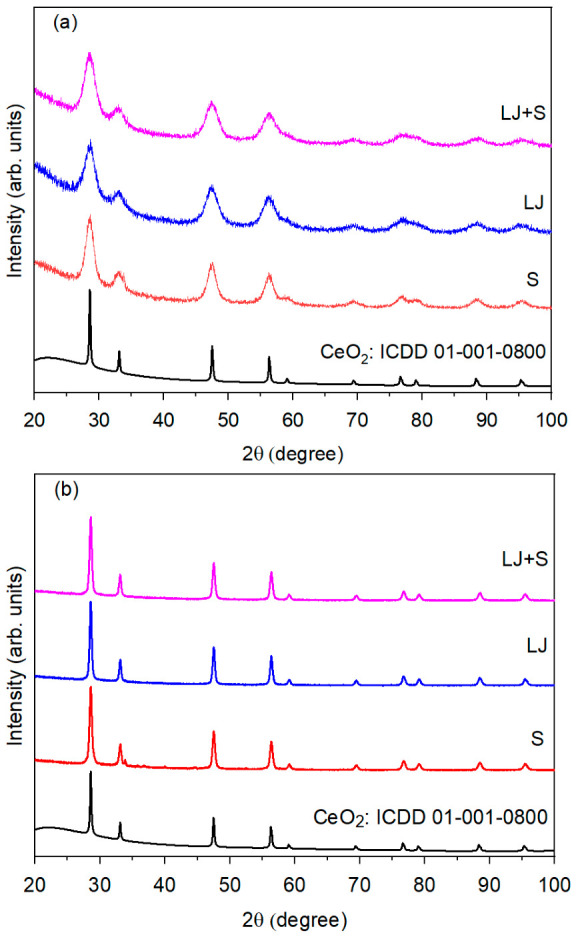
X-ray diffraction patterns of the three samples (S, LJ, and LJ+S) after heat treatment at (**a**) 350 °C and (**b**) 600 °C, with a reference CeO_2_ diffraction pattern (ICDD 01-001-0800) included for comparison.

The XRD patterns for the three samples treated at 350 °C and 600 °C are shown in Figure 3a and Figure 3b, respectively. A comparative analysis reveals significant structural evolution as a function of the calcination temperature. In all samples (S, LJ, and LJ+S), the diffraction peaks align with the characteristic indices of the face-centred cubic fluorite-type structure [30] of CeO_2_ (ICCD 01-001-0800) shown in Figure 3b.

For the first calcination temperature (350 °C), the diffraction patterns exhibit broad and poorly defined peaks, indicating low crystallinity and/or very small crystallite size, with a possible contribution from amorphous material. In contrast, increasing the thermal treatment to 600 °C (Figure 3b) resulted in a dramatic improvement in the diffraction profiles. The peaks for all three samples remain centered at the same 2θ positions but appear significantly narrower and sharper. This transformation reflects enhanced crystallinity and the promotion of crystallite growth as the thermal energy facilitates the reorganization of the Ce-O lattice and the removal of surface defects [31].

A notable deviation was observed in the S sample at 600 °C, where a new diffraction peak (2θ ≈ 34°) emerged that was not present at the lower temperature. This auxiliary peak indicates the formation of a secondary crystalline phase. Given the synthesis conditions, specifically the use of NaOH for pH adjustment and the carbon-rich nature of the sucrose precursor, this phase is tentatively identified as sodium bicarbonate (NaHCO_3_). The position of this secondary reflection aligns with the most intense diffraction peaks reported for NaHCO_3_ in the literature [32]. At 350 °C, this phase was likely either amorphous or masked by the broad reflections of the poorly crystallized CeO_2_ matrix [33,34]. These results demonstrate that the thermal treatment at 600 °C is significantly more effective than 350 °C for producing highly crystalline CeO_2_ nanostructures.

To quantify the influence of the various green precursors on the resulting nanostructure, the average crystallite size (*D*) of the samples calcined at 600 °C was estimated using the Scherrer equation [35]:

(2) $$D=\frac{K\;\lambda }{\beta \;cos\theta }$$

where *K* is the shape factor (0.94), *λ* is the X-ray wavelength (1.5406 Å), *β* is the full width at half maximum (FWHM) of the (111), (200), and (220) planes, and *θ* is the Bragg angle.

The average crystallite sizes ranged from 15.2 nm to 17.2 nm. The LJ sample yielded the largest crystallites (17.2 nm), while the addition of sucrose in the LJ+S (15.8 nm) and S (15.2 nm) samples resulted in a slight reduction in size. This narrow distribution suggests that while the lemon juice extract facilitates crystallite growth, sucrose acts as a mild capping agent that stabilizes the nanostructure. This aligns with previous reports on carbohydrate sugars and sucrose as green capping/stabilizing agents for CeO_2_ nanoparticles [25,36].

### 3.3. Raman Spectroscopy

**Figure 4:**
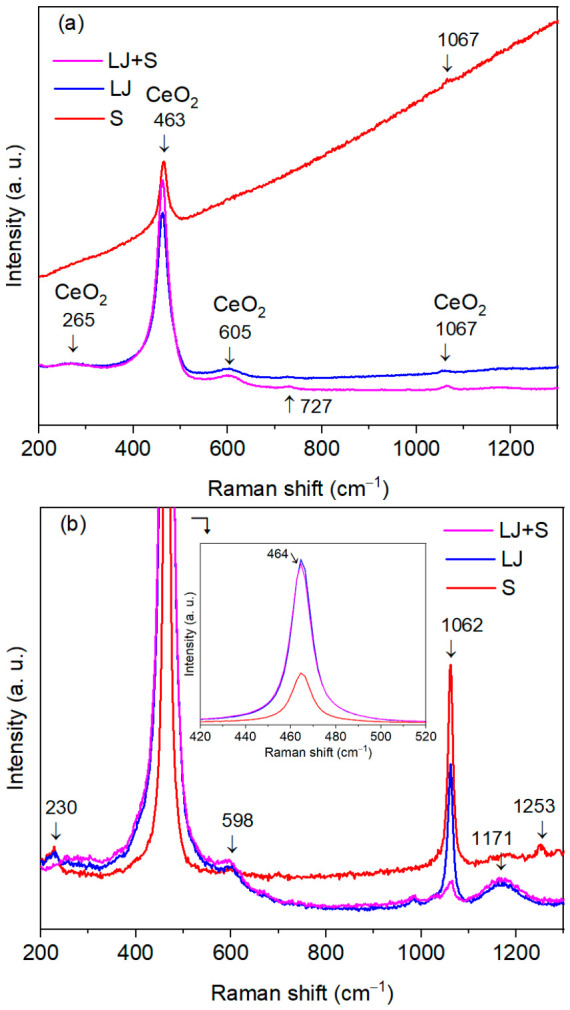
Raman spectra of the three samples (S, LJ, and LJ+S) under heat treatment at (**a**) 350 °C and (**b**) 600 °C.

Raman spectroscopy was employed to investigate the structural properties and phase purity of the synthesized CeO_2_ nanoparticles. Figure 4 displays the Raman spectra for the S, LJ, and LJ+S samples calcined at 350 °C and 600 °C.

Raman spectra for the three samples calcined at 350 °C are presented in Figure 4a. The spectra are dominated by a prominent peak at 463 cm^−1^, which corresponds to Ce–O lattice vibrations, specifically the F_2g_ mode of the cubic fluorite phase of CeO_2_ [37]. Additionally, weaker bands are observed at 265, 605, and 1067 cm^−1^. The band at 265 cm^−1^ is commonly ascribed to a second order, doubly degenerate transverse acoustic (2TA type) vibration of the CeO_2_ lattice, whereas the 605 cm^−1^ feature is attributed to a defect related longitudinal optical (LO) mode associated with oxygen vacancy formation and the accompanying Ce^4+^/Ce^3+^ redox process [38]. The band around 1067 cm^−1^ is assigned to a higher order overtone/combination vibration of the cubic fluorite lattice, in line with previous reports on nanostructured CeO_2_ [39]. A weak additional feature at 727 cm^−1^ is observed exclusively in the LJ+S sample. Since this band is not characteristic of fluorite CeO_2_, it most likely arises from a minor secondary phase. The corresponding XRD pattern at 350 °C (Figure 3a) does not show extra crystalline reflections, suggesting that this phase is either amorphous or is masked by the broad reflections of the poorly crystallized CeO_2_ matrix. Notably, the S sample also displays a pronounced elevation of the Raman baseline across the entire spectral range. This behaviour is characteristic of fluorescence, most likely originating from residual carbonaceous species that absorb the excitation light and re-emit at longer wavelengths, thereby superimposing a broad background on the Raman spectrum. This fluorescent contribution partly obscures weaker vibrational modes and complicates the reliable identification of low intensity Raman bands [40].

After thermal treatment at 600 °C, Raman spectra were again recorded for the three samples (Figure 4b). The characteristic bands of CeO_2_ at approximately 265, 463, 605, and 1067 cm^−1^ are still observed, confirming that the fluorite crystalline phase is preserved after this calcination. The sharp and intense F_2g_ mode at 463 cm^−1^ remains the dominant feature, consistent with the formation of nanometric CeO_2_ particles. In addition, a distinct band appears at 1171 cm^−1^, which is only detected in the samples containing lemon juice (LJ and LJ+S) and is absent in the sucrose only sample (S). The band at 1171 cm^−1^ is characteristic of CeO_2_ and has been assigned to the second order (2LO) vibration associated with the bulk structure of the fluorite lattice [39]. This feature is reported to remain essentially unchanged under reduction treatments and is therefore frequently used as an internal reference in Raman analyses of ceria. Its presence in the LJ and SLJ samples indicates that the CeO_2_ crystalline framework remains well defined after calcination at 600 °C [41].

A further Raman band emerges at 1253 cm^−1^ exclusively in the S sample after heat treatment at 600 °C, which is not present in the corresponding spectrum at 350 °C. Since this band is not typical of CeO_2_, it can be associated with a secondary phase and is plausibly linked to the additional diffraction peak observed in the XRD pattern of the S sample at 600 °C, tentatively assigned to sodium bicarbonate (NaHCO_3_). Thomas Rainer [42] reported that the Raman spectrum of NaHCO_3_ exhibits several characteristic vibrational modes, including an intense band at about 1045 cm^−1^ and a weaker band near 1266 cm^−1^. The strong 1045 cm^−1^ band can overlap with the CeO_2_ s order signal close to 1060 cm^−1^ and thus remain unresolved in our spectra, whereas the band detected at 1253 cm^−1^ in the S sample can be correlated with the weaker NaHCO_3_ vibration typically reported around 1260 cm^−1^, supporting the presence of NaHCO_3_ as a secondary phase even when its most intense Raman features are not clearly separated from those of CeO_2_.

### 3.4. SEM Analysis

**Figure 5:**
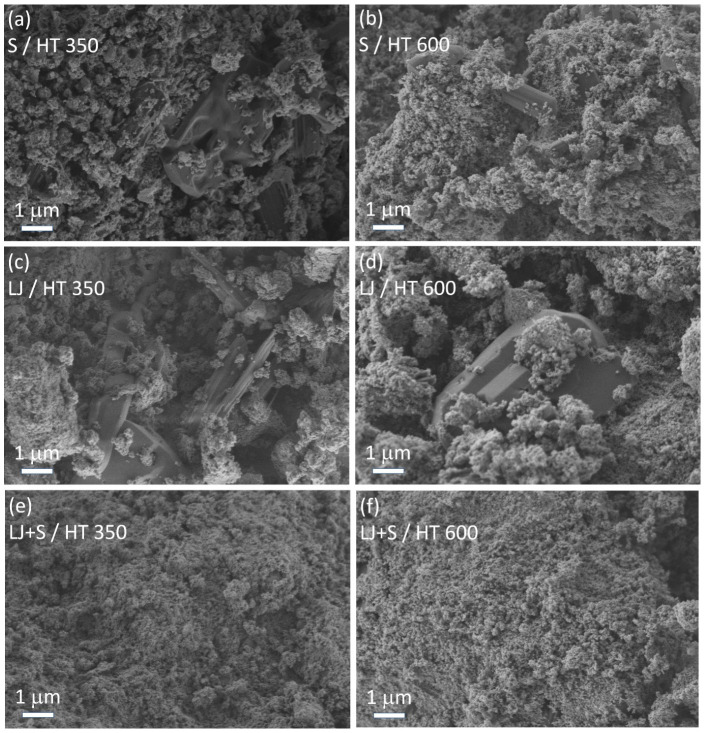
SEM micrographs of CeO_2_ samples synthesized using sucrose (S), lemon juice (LJ), and their combination (LJ+S), after heat treatment at 350 °C and 600 °C: (**a**) S/HT350, (**b**) S/HT600, (**c**) LJ/HT350, (**d**) LJ/HT600, (**e**) LJ+S/HT350, and (**f**) LJ+S/HT600.

SEM was used to evaluate the morphological evolution of the CeO_2_ nanoparticles synthesized via three distinct green routes (S, LJ, and LJ+S) under controlled thermal treatments at 350 °C and 600 °C, as shown in Figure 5.

Regarding the micrograph analysis of the samples calcinated at 350 °C, the morphology at this stage is characterized by large, dense clusters, likely due to the presence of residual amorphous organic matter that prevents the formation of well-defined primary nanoparticles. A notable feature in the S and LJ samples is the presence of large structures (Figure 5a,c) resulting from the coalescence and agglomeration of particles [43]. Interestingly, these large formations persisted even after increasing the thermal treatment to 600 °C (Figure 5b,d). However, the LJ+S samples presented a distinct exception (Figure 5e,f). Although some degree of coalescence is still observed, the nanoparticles exhibit significantly improved dispersion and a more refined morphology, characterized by a homogeneous distribution of predominantly spherical particles. This enhanced morphological outcome is attributed to the synergistic stabilizing effects of the dual precursor system. The citric acid and various phytochemicals in the lemon juice act as effective chelating agents, which help stabilize the initial nuclei and prevent early stage coalescence [44]. Meanwhile, sucrose acts as both a structure-directing and capping agent, promoting the formation of smaller and more uniformly dispersed nanoparticles by limiting agglomeration and enhancing colloidal stability [25].

Under the acidic conditions provided by the citric acid present in lemon juice, sucrose can hydrolyze into glucose and fructose. Upon heating, glucose and fructose may undergo oxidation and thermal degradation reactions, yielding oxygenated intermediates rich in hydroxyl (–OH) and carboxyl (–COOH) functional groups. These species can coordinate Ce^3+^ ions derived from Ce(NO_3_)_3_·6H_2_O and contribute to the formation of an organic–inorganic network, playing a role analogous to that of polyols such as ethylene glycol in polymeric precursor methods. During calcination, the organic matrix decomposes while the coordinated cerium species undergo condensation and crystallization, leading to the formation of stoichiometric CeO_2_ nanoparticles. Although CeO_2_ formation occurs in all formulations, the combined presence of lemon juice and sucrose appears to provide improved control over nanoparticle development, resulting in enhanced dispersion and morphological homogeneity [45,46,47].

**Figure 6:**
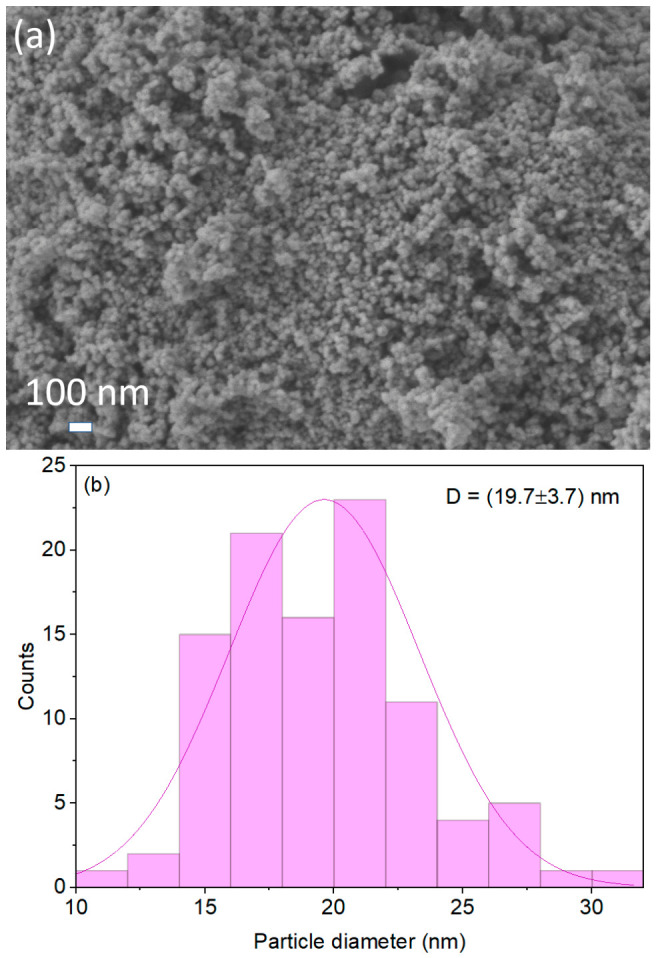
(**a**) High-magnification SEM micrograph of the CeO_2_ sample synthesized using sucrose and lemon juice (LJ+S), after heat treatment at 600 °C; (**b**) Particle size distribution obtained from measurements of 100 nanoparticles. The pink columns and solid curve represent the experimental data histogram and the corresponding Gaussian fit, respectively.

To further examine the particle morphology and size distribution, a higher-magnification micrograph of the LJ+S sample and the corresponding particle size distribution histogram are presented in Figure 6.

Particle size analysis was carried out using ImageJ software (version 1.53m). The diameters of 100 nanoparticles randomly selected from different regions of the micrograph were measured to obtain a representative size distribution. As the particles displayed an approximately spherical morphology, their size was determined from a single diameter measurement. The resulting average particle diameter was (19.3 ± 3.7) nm.

To better contextualize the physicochemical characteristics of the CeO_2_ nanoparticles synthesized in this work and highlight the novelty of the proposed green synthesis route, Table 2 compares the present approach with selected studies reporting the green synthesis of CeO_2_ nanoparticles using different natural extracts. The comparison includes the bio-based agent, synthesis method, crystallite size, particle size, and particle morphology. This overview provides a broader perspective on the structural and morphological characteristics of CeO_2_ nanoparticles obtained through various green synthesis strategies and highlights the position of the proposed lemon juice–sucrose route within the current state of the art.

### 3.5. Antibacterial Activity

**Figure 7:**
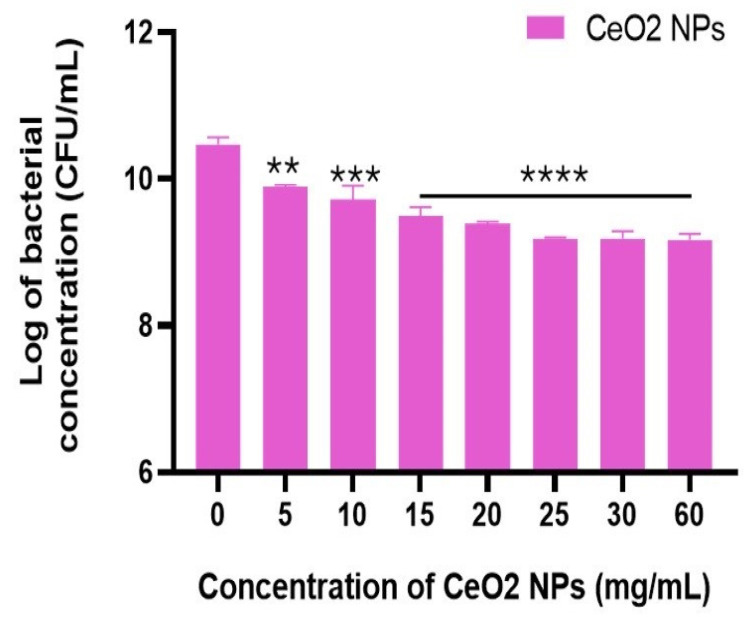
Logarithm of bacterial concentration after 24-h exposure to increasing concentrations of CeO_2_ nanoparticles (LJ+S sample calcined at 600 °C). Results are expressed as mean ± standard deviation (SD). Statistically significant differences compared to the untreated control group are indicated by asterisks (**** *p* < 0.0001, *** *p* < 0.001 and ** *p* < 0.01).

The antibacterial effect of the biosynthesized CeO_2_ nanoparticles (LJ+S sample calcined at 600 °C) against *Staphylococcus aureus* (*S. aureus*) was evaluated by monitoring the logarithmic reduction in CFU/mL after 24 h of exposure. The LJ+S sample was chosen based on its superior physicochemical characteristics, including a more homogeneous morphology, reduced presence of large secondary structures, and higher phase purity, as evidenced by SEM, XRD, and Raman analyses. As illustrated in Figure 7, the nanoparticles exhibited a clear dose dependent inhibitory effect.

A reduction in bacterial viability was observed at 5 mg/mL (** *p* < 0.01), indicating the onset of antibacterial activity. This effect became more pronounced at 10 mg/mL (*** *p* < 0.001), confirming a concentration-dependent response. Highly significant antibacterial activity (**** *p* < 0.0001) was detected at concentrations ≥ 15 mg/mL. Notably, the antibacterial effect reached a plateau at approximately 25 mg/mL, beyond which further increases in CeO_2_ NP concentration did not result in a proportional reduction in CFU counts, suggesting that the maximum effective dose under the tested conditions had been attained [53].

Several physicochemical factors may contribute to this observed saturation. A primary explanation involves the concentration dependent aggregation of CeO_2_ nanoparticles. At elevated concentrations, the increase in particle-particle collisions often leads to the formation of larger agglomerates, which significantly reduces the effective surface-area-to-volume ratio available for interaction with the bacterial cells. This reduction in active surface area diminishes the density of reactive sites available for oxidative stress induction and direct membrane disruption, two primary drivers of nanoceria toxicity [54,55]. Furthermore, the observed tendency of the nanoparticles to sediment at higher concentrations may limit the bioavailability of the particles in the liquid phase, thereby reducing the effective contact frequency with the suspended bacteria.

The pH of the nanoparticle suspensions was also monitored after 24 h of incubation under the same conditions used for the antibacterial assays. The untreated bacterial control exhibited a pH of 6.68, while the CeO_2_-treated bacterial suspensions showed pH values ranging from 6.49 to 7.38, depending on nanoparticle concentration. These results indicate that all conditions remained close to neutral pH throughout the experiment. Therefore, the observed reduction in bacterial viability cannot be attributed to major pH-induced stress and is more likely associated with the intrinsic antibacterial properties of the CeO_2_ nanoparticles.

Several mechanisms may contribute to the antibacterial activity observed for the CeO_2_ nanoparticles. Although the present study did not aim to elucidate the bactericidal mechanism in detail, previous reports suggest that CeO_2_ nanoparticles can affect bacteria through multiple, possibly simultaneous, pathways. One of the main proposed mechanisms involves direct interaction between nanoparticles and the bacterial cell envelope [56]. Due to electrostatic and physicochemical interactions, CeO_2_ nanoparticles may adsorb onto the bacterial surface, promoting close contact with the cell wall and membrane. This interaction can disturb membrane organization, alter permeability, impair ion transport, and affect nutrient exchange between the bacterial cell and the surrounding medium. In addition, cerium species may interact with membrane-associated proteins, including thiol-containing groups, as well as transporters and porins, further compromising bacterial homeostasis [57]. Another important contribution may arise from the redox activity of CeO_2_ nanoparticles. The reversible Ce^3+^/Ce^4+^ transition, together with the presence of oxygen vacancies, can promote catalytic reactions associated with the generation of reactive oxygen species (ROS), including superoxide radicals, hydroxyl radicals, and hydrogen peroxide, depending on the physicochemical properties of the nanoparticles and the surrounding environment [18,58]. These ROS can induce oxidative stress and damage essential bacterial components such as membrane lipids, proteins, polysaccharides, proteins, and nucleic acids, ultimately leading to loss of cellular integrity and bacterial death [59]. Furthermore, oxygen vacancies are recognized as key active sites that enhance the catalytic activity of CeO_2_ nanoparticles and may contribute to ROS-mediated antibacterial effects. Particle size, morphology, surface defects, and oxygen vacancy concentration have also been reported to influence antibacterial performance by modulating surface reactivity, bacterial contact, and ROS generation [60].

Therefore, the reduction in viable *S. aureus* cells observed in this study is likely associated with the combined contribution of nanoparticle–cell envelope interactions and CeO_2_-mediated redox activity.

In summary, the CeO_2_ nanoparticles synthesized via the green LJ+S route demonstrate robust antibacterial efficacy against *S. aureus*. While aggregation and sedimentation present inherent limitations to dose escalation, the high activity observed at relatively low concentrations confirms the potential of these biosynthesized nanoparticles as effective agents for biomedical coatings or antibacterial formulations. It should be noted that this study represents an initial evaluation of antibacterial performance, focused exclusively on *S. aureus* as a model Gram-positive bacterium. Future work will extend this assessment to a broader range of clinically relevant bacterial strains, including Gram-negative species, and will further investigate the underlying antibacterial mechanisms through complementary physicochemical and biological analyses, including ROS quantification, membrane integrity assessment, and bacterial morphology characterization.

## 4. Conclusions

This work demonstrates a convenient green synthesis route for CeO_2_ nanoparticles using lemon juice, sucrose, and their combination as bio-based chelating, capping, and stabilizing agents. Thermal, structural, and vibrational analyses collectively show that calcination at 600 °C is essential to obtain highly crystalline cubic fluorite CeO_2_, while the precursor chemistry decisively governs phase purity and morphology.

XRD and Raman spectroscopy confirm that all samples exhibit nanocrystalline CeO_2_ with average crystallite sizes in the 15–17 nm range, whereas sucrose-only processing promotes the formation of a NaHCO_3_ secondary phase at high calcination temperature. The combined lemon juice/sucrose system effectively suppresses this secondary phase and stabilizes the fluorite lattice. SEM further reveals that this dual precursor route mitigates the formation of large coalesced aggregates, offering homogeneous, well dispersed, and spherical nanoparticles that are not achieved with either lemon juice or sucrose alone.

Biological assays against Staphylococcus aureus demonstrate a clear dose-dependent antibacterial response for the LJ+S-derived CeO_2_ nanoparticles, with significant reductions in CFU/mL at concentrations ≥5 mg/mL and a pronounced bactericidal effect up to a saturation regime around 25 mg/mL. The high activity at relatively low doses, combined with the green synthesis route and favorable nanoscale morphology, highlights these biosynthesized CeO_2_ nanoparticles as promising candidates for integration into antimicrobial coatings and biomedical applications.

## Figures and Tables

**Table 1:** Initial and final pH values, along with the corresponding visual evolution (color and viscosity changes) of the reaction mixtures during the sol–gel–derived synthesis of CeO_2_ nanoparticles for the different compositions (S, LJ, and LJ+S).

Sample	pH (Initial–Final)	Visual Evolution
S	4.8–11.2	Transparent → golden → gray coloration with increased viscosity
LJ	0.98–7.6	Increase in yellow coloration and viscosity
LJ+S	1.1–5.3	Increase in yellow coloration and viscosity

**Table 2:** Comparison of CeO_2_ nanoparticles synthesized using different green routes, including crystallite size, particle size, morphology, and key distinguishing features.

Bio-Based Agent	Synthesis Method	Crystallite Size (nm)	Particle Size (nm)	Morphology	Ref.
*Acacia concinna* fruit extract	Sol-gel	22.7	-	Porous network of roughly spherical nanoparticles; uniform distribution with limited agglomeration	[18]
*Citrus nobilis* peel extract	Co-precipitation	5.1	25	Spherical to quasi-spherical nanoparticles; uniform distribution with moderate agglomeration	[19]
*Olea europaea* leaf extract	Precipitation	6	24	Highly homogeneous distribution of spherical nanoparticles	[48]
*Magnolia kobus* leaf extract	Precipitation	20	50	Nearly spherical, well-dispersed nanoparticles	[49]
*Solanum nigrum* leaf extract	Precipitation	10.08	20	Spherical to quasi-cubic, uniformly dispersed nanoparticles with reduced agglomeration	[50]
*Crocus sativus* by-product extract	Precipitation	16.71	-	Spherical nanoparticles with a homogeneous distribution	[51]
*Abelmoschus esculentus* fruit extract	Precipitation	30	36	Spherical nanoparticles with a homogeneous distribution	[52]
Lemon juice + sucrose	Sol gel based	15.8	19.7	Quasi-spherical nanoparticles with improved dispersion and homogeneity	This work

## Data Availability

The original contributions presented in this study are included in the article. Further inquiries can be directed to the corresponding author.
